# Internet Gambling Among High School Students in Hong Kong

**DOI:** 10.1007/s10899-013-9413-6

**Published:** 2013-11-05

**Authors:** Irene Lai Kuen Wong, Ernest Moon Tong So

**Affiliations:** 1 Department of Applied Social Sciences, Hong Kong Polytechnic University, Hung Hom, Kowloon, Hong Kong; 2 Department of Sociology, University of Hong Kong, Pokfulam Road, Hong Kong

**Keywords:** Internet gambling, Problem gambling, Addiction, Adolescent students

## Abstract

The study investigated Internet gambling involvement and pathological gambling among Hong Kong adolescents aged 12–19 years. The diagnostic and statistical manual (4th edition) multiple response format for juveniles (DSM-IV-MR-J) (Fisher in J Gambl Stud 16:253–273, 2000) was filled by 1,004 students (597 boys, 407 girls) recruited by random selection of classes. The response rate was 86.6 %. Results indicate that more respondents participated in land-based gambling than Internet gambling (63.5 vs. 3.5 %) but online gamblers are 1.5 and 3.2 times more likely to develop pathological and at-risk gambling than non-Internet gamblers. Using the DSM-IV-MR-J criteria, 5.7 and 22.9 % of the Internet gamblers could be classified as at-risk gamblers and pathological gamblers, respectively. Majority (94.3 %) wagered online at home, and 91.4 % made their first bet before 18 years. Many perceived Internet gambling as a trendy (71.4 %) and safe entertainment (54.3 %). Problematic Internet gambling was significantly associated with the male gender, school grades, online gambling frequency, amount wagered and a gambling family environment. Survey results have implications for gambling research and preventive programs.

## Introduction

Internet gambling is a fast expanding industry which was expected to generate global gross revenues of US$21 billion from players worldwide in 2008 (American Gaming Association 2007). There has been explosive growth of online gambling sites since the first site was launched in the mid 1990s. Gamcare (2007) estimated that 3,000 gambling sites would be available by 2007. With the phenomenal increase of gambling sites, more people especially youth will engage in the activity (Matthews et al. 2009; Romer 2010; Woolley 2003).

The first 1999 British prevalence survey revealed that none of the respondents aged 15–19 years had gambled online (Griffiths 2001). Few years later, Wood et al. (2007) found a 5 % online poker playing rate in a United Kingdom university student sample. In Canada, 3.7 % of Quebec high school teenagers staked online in the previous year (Chevalier et al. 2003), while 11.7 % of 631 Ontario underage students had wagered on the Internet (Ladouceur et al. 2005). Approximately 30–40 % of Canadian youth might be playing on the practice sites (Byrne 2004; Derevensky and Gupta 2007). Romer (2010) noted that monthly Internet gambling shot up from 4.4 % in 2008 to 16 % in 2010 among male United States youngsters aged 18–22 years.

There have been growing concerns about Internet gambling among adolescents who are frequent Internet users (Derevensky and Gupta 2007; Ladouceur et al. 2005; Wong 2010a). They may be lured by the pop up gambling advertisements, offers of gifts and free play, tempting easy win messages, thrill of many online games, and visually exciting graphics and photos presented with the games (Derevensky and Gupta 2007). Transfer of gambling payments may hamper some of the underage teens to bet online. However, payment restrictions are no longer a barrier when payment technologies (e.g., PayPal and Firepay systems) are widely accessible or adult assistance is available. Internet gambling could be potentially more tempting and addictive than offline gambling due to convenience, accessibility, affordability, anonymity and interactivity (Griffiths 2003; Wood et al. 2007).

Recent available data suggest the prevalence rates of pathological gambling are higher among Internet gamblers than offline players. The gambling disorder is characterized by dependence, loss of control and disruptions to significant areas of a gambler’s life (American Psychiatric Association 1994). A study of 1,356 United States college students (Petry and Weinstock 2007) showed that 61.6 % of regular Internet gamblers were pathological gamblers, compared with 23.9 % of infrequent Internet gamblers and 5.0 % of non-Internet gamblers. The rate of problem gambling among 465 Canadian youth online gamblers was 2.7 times higher than the non-Internet gamblers (McBride and Derevensky 2012). A United Kingdom survey revealed 37 % of 127 United Kingdom university student Internet gamblers had gambling problems in their lifetimes (probable pathological gamblers, 19 %; potential pathological gamblers, 18 %) (Matthews et al. 2009). Olason et al. (2011) reported only 1.1 % of Iceland adolescent offline gamblers showed signs of problem gambling, but 7.7 % of online gamblers were identified as problem gamblers. These studies indicate Internet gamblers were more susceptible to problem gambling than offline gamblers (Griffiths and Barnes 2008).

There is little research on Internet gambling among Chinese adolescents. Approximately 2–4.6 % of high school students (grade 7–12) in Hong Kong gambled online in the past year (Hong Kong Polytechnic University 2002; University of Hong Kong 2005; Wong 2010b). In Macau, 5.2 % of high school students (grade 9–12) wagered at online casinos (University of Macau 2003). Wong (2010a) reported that 6.6 % of 422 Macau high school students (grade 7–12) gambled online, and 25 and 10.7 % could be designated as pathological and at-risk gamblers, respectively.

To fill the research gap, this study aimed to gauge and compare prevalence estimates of offline and Internet gambling participation and pathological gambling among Hong Kong high school students. The legal age of gambling in Hong Kong is 18 years. The survey also examined adolescents’ perceptions of Internet gambling, and their reasons for participation and non-participation. The last aim was to identify demographic and behavioral correlates of problematic Internet gambling. It was hypothesized that the rates of pathological and at-risk gambling would be higher among online gamblers than offline gamblers. We expect online gamblers’ perceptions of Internet gambling would be more positive than those who did not participate in the activity. Further, it was hypothesized that boys would be more vulnerable to Internet gambling involvement and problem gambling than their female counterparts. The research findings will shed light on future research and preventive measures.

## Methods

### Procedures

The study was conducted in 2010. First, an invitation letter outlining the survey objectives and procedures were mailed to five high schools randomly chosen from a school list published by the education authority. With the principals’ approval from four schools, a trained researcher administered the questionnaires to students in class. Two classes of 7–12 grades were randomly selected from these high schools. The survey purposes and procedures were fully clarified before seeking the students’ informed consent to participate. Participation was voluntary and anonymous. A questionnaire was distributed to 1,160 students. Only 1,004 usable questionnaires were returned and used in data analysis. The response rate was 86.6 %.

### Participants

Among the participants, 597 (59.5 %) were boys, 407 (40.5 %) were girls; 602 (60 %) were junior graders, 402 (40 %) were senior graders. Overall, one-third (33.6 %) were between 12–13 years, 40.6 % (n = 408) were 14–15 years, and 25.7 % (n = 258) were 16–19 years. The mean age was 14.7 years (SD = 2.1).

### Instruments

A standardized self-administered questionnaire was designed to collect information. There are five parts in the survey questionnaire:

- Demographic questions on age, gender, school grades, and sources of pocket money;
- A two-item Internet Usage Questionnaire asks the participants if they were Internet users in the last 12 months, and how many hours they usually spent on the Internet each day.
- An Internet Gambling Behavior Questionnaire investigates why, where, how much and how frequent the participants gambled online in the past year. The online gamblers had to report their preferred forms of Internet gambling and methods to transfer payment. They were asked when they first wagered on the Internet, and if their peers and family members had gambled online in the preceding year. Lastly, they had to answer if they were living with family members who had gambling problems. Students were also asked if they had gambled offline during the previous year so that comparison between online and offline gambling behavior can be made.
- Based on Griffiths’ study (2001), a 7-item perceptions of Internet gambling questionnaire (PIGQ) was designed to examine the participants’ perceptions of Internet gambling activities on a four-point scale (0—strongly agree, 1—agree, 2—disagree, 3—strongly disagree). Questions focus on whether Internet gambling may cause harmful consequences (item 7), and would potentially be more addictive than traditional gambling (item 6). Other questions include if Internet gambling was viewed as a less regulated (item 5) but trendy activity (item 1), a safe gambling option (item 2), a healthy entertainment (item 3) with an opportunity to win money. (item 4). Total scores range from 0 to 21. Higher scores suggest more positive attitudes towards Internet gambling. The questionnaire is reliable (Cronbach’s α = 0.7). Factor analysis (Harman 1967) showed satisfactory construct validity. A two factor solution explaining 58.4 % of the total variance was generated from a principal component analysis. The first factor (explaining 38.9 % of variance) is composed of item 1, 2, 3 and 4. It could be labeled as “enticement of Internet gambling”. Factor 2 (explaining 19.5 % of variance) consisting of item 5, 6 and 7 could be labeled as “risks of Internet gambling”. In brief, this short questionnaire is useful for assessing adolescents’ perceptions of Internet gambling in a user friendly and time-effective manner.
- The diagnostic and statistical manual of mental disorders (DSM-IV; American Psychiatric Association 1994) multiple response format for juveniles (DSM-IV-MR-J) (Fisher 2000) assesses adolescent online and offline gambling problems in the last year. Survey participants were asked to complete the DSM-IV-MR-J twice, first for involvement in traditional forms of gambling and then for Internet gambling. The 12-item DSM-IV-MR-J is a reliable (Cronbach’s α = 0.75) and valid gambling screen (Fisher 2000). Nine distinct categories of pathological gambling are identified, namely preoccupation, tolerance, withdrawal, loss of control, escape, chasing losses, lying, illegal acts to pay for gambling, and disrupted relationships. Endorsement of four or more of the nine categories suggests probable pathological gambling, endorsement of 2–3 categories indicates at-risk gambling, and identification of 0–1 category is a sign of social gambling.

### Statistical Analysis

The quantitative data generated from the survey questionnaires were converted to SPSS Statistics 18.0 for analysis using distributions of frequency, cross-tabulations, Chi square and the Mann–Whitney U tests (where suitable). To identify the correlates of problematic Internet gambling, the non-parametric Mann–Kendall Tau-b test was used as there were only 35 Internet gamblers.

## Results

### Participation and Addiction in Offline Gambling

For the entire sample (n = 1,004), 63.5 % reported gambling offline during the previous 12 months. Among these 638 offline gamblers, 3.9 % (n = 25) could be classified as probable pathological gamblers (endorsed four or more categories), and 7.1 % (n = 45) were at-risk gamblers (endorsed 2–3 categories) using the DSM-IV-MR-J criteria.

### Internet Gambling Involvement and Addiction

A great majority of the participants (96.9 %, n = 973) had access to the Internet in the preceding year. Many (70.8 %) spent at least 2 h every day on the Internet playing electronic games (82.3 %), searching for information (51.8 %), social networking (32.5 %) and sending messages (14.5 %). On average, the students spent 2.71 h (SD = 1.8) on the Internet.

Most (73.3 %) opined that gambling information was widely available on the Internet, but only 3.5 % (n = 35) gambled online with money in the past year. A higher proportion (63.5 %) reported gambling offline. One teen gambled exclusively online, while 34 Internet gamblers (97.1 %) reported gambling both online and offline. In line with previous data (McBride and Derevensky 2012; Wardle and Griffiths 2011; Wood and Williams 2011), “Internet gamblers” refer more to players who have wagered online and offline. A great majority (82.4 %) reported gambling more frequently on the Internet, whereas 17.6 % gambled less frequently online compared with offline gambling.

Using the DSM-IV-MR-J criteria (Fisher 2000), 10 Internet gamblers exhibited symptoms of problematic gambling (endorsed two or more categories). They constituted 1 % of the entire sample (n = 1,004), and 28.6 % of the online gamblers (n = 35). Among the online gamblers, two (5.7 %) could be identified as probable pathological gamblers, and eight (22.9 %) were at-risk gamblers. Table 1 provides a summary of the offline players and the Internet gamblers.

**Table 1:** Internet gamblers and offline gamblers

Problem gambling severity (DSM-IV-MR-J criteria)	Internet gamblers (N = 35)		Offline gamblers (N = 638)	
	N	%	N	%
Social gamblers (endorsed 0–1 category)	25	71.4	568	89
At-risk gamblers (endorsed 2–3 categories)	8	22.9	45	7.1
Pathological gamblers (endorsed 4 or more categories)	2	5.7	25	3.9
Total	35	100.0	638	100.0

### Gender Difference in Internet Gambling and Problematic Gambling

For the entire sample (n = 1,004), 2.9 % of boys (n = 29), and 0.6 % of girls (n = 6) reported gambling online during the previous 12 months. Boys were 4.8 times more likely than girls to wager online.

All the eight at-risk Internet gamblers were boys (100 %). One of the two probable pathological online gamblers was a girl (50 %), the other gambler was a boy (50 %). Pathological and at-risk Internet gamblers were more likely to be boys (90 %) than girls (10 %).

### Symptoms of Problematic Internet Gambling

The ten problematic Internet gamblers (endorsed 2 or more categories) reported many symptoms of problem gambling including lying (70 %), damaged significant relationships (60 %), chasing losses (50 %), escape from problems (40 %), tolerance (40 %), illegal acts to finance Internet gambling (30 %), withdrawal (20 %), losing control (20 %) and preoccupation with gambling (10 %).

### Age of Internet Gamblers

Most of the Internet gamblers (88.6 %, n = 31) were below 18 years, only four (11.4 %) aged 18–19 years. The mean age was 14.5 years (SD = 1.8). Majority (77.4 %, n = 24) of these underage online gamblers were social or recreational gamblers but seven (22.6 %) had Internet gambling problems (three at-risk gamblers and four pathological gamblers). Two (50 %) of the adult online players (18 years or above) were social gamblers, another two (50 %) showed signs of a gambling problem (one at-risk gambler and one pathological gambler).

### School Grades of Internet Gamblers

Twenty-one Internet gamblers (60 %) were in the junior grades of 7–9, 14 (40 %) were senior graders of 10–12. All the junior graders (n = 21) played at free trial sites in the previous 12 months, but only four (28.6 %) senior graders played at these sites. The senior graders preferred wagering money on the paid sites (85.7 %), and would resort to playing at the practice sites only when they had no money to stake with.

### Internet Gambling Preferences

The preferred forms of online gambling within the whole sample (n = 35) were mahjong (54.3 %), poker cards (45.7 %), casino dice games (31.4 %), soccer betting (20 %), lotteries (17.1 %) and horse racing (11.4 %).

Problematic Internet gamblers favored soccer betting (60 %), casino dice games (40 %) and horse races (40 %), whereas non-problematic Internet gamblers chose mahjong (64 %), card games (56 %) and casino dice games (28 %).

### Early Internet Gambling Initiation

Only three Internet gamblers (8.6 %) first wagered online at 18 years or older, majority (91.4 %, n = 32) began the activity at a very young age, including 60 % (n = 21) at 11 years or below, and 40 % between 12–17 years.

### Internet Gambling Frequency and Money Wagered

Many Internet gamblers (80 %) played 1–3 times a week, 14.3 % gambled 4–6 times weekly, 5.7 % wagered at least once each day. Problematic Internet gamblers bet online significantly (mean = 3 times weekly) more frequent than social gamblers (mean = 1.1 times weekly) (Z = −3.375, *p* < 0.001).

Most (82.9 %) bet less than HK$100 a week, 14.3 % wagered HK$101–$200, and 2.9 % staked more than HK$1,000. Problematic Internet gamblers wagered significantly more money each week (mean = HK$250) than social gamblers (mean = HK$96.5) (Z = −3.795, *p* < 0.001).

### Source of Gambling Money and Payment Methods

A great majority (94.3 %) used the pocket money provided by their parents to gamble online in the preceding year. Most (94.3 %) wagered online at home, three (8.6 %) bet at friends’ residence, and two (5.7 %) played at school. Many started playing first on the “free” sites with money or free chips offered by the site operators (71.4 %) before gambling at the paid sites. Many underage gamblers were helped to bet online by using adults’ Internet gambling accounts (41.9 %), and visa or credit cards (32.3 %). Assistance provided by friends (10.3 %) and family members (42.8 %) played a vital role in transferring gambling payments.

### Reasons for Internet Gambling

The enticement of the activity for Internet problematic gamblers (endorsed two or more categories) was convenience (70 %), anonymity (50 %), flexibility to play different games simultaneously (50 %) and attractive gambling advertisements (40 %). They were also influenced by peer encouragement (40 %) and family members who gambled regularly (50 %) either online or offline. Many (70 %) had peers who also gambled online. More online problematic gamblers reported having family members being distressed with gambling problems than social gamblers (60 vs. 16 %).

Social gamblers were lured by attractive games which helped relieving boredom and killing time (48 %), offers of money and free chips provided by the site operators (37.1 %), peer influence (28 %), colorful advertisements (20 %), and acceptance of low wagers (12 %).

### Reasons for Non-participation

The reasons for making no attempts to gamble online given by 969 students were lack of interest (55.7 %) and knowledge about the activity (18.8 %), being unfamiliar with the gambling sites (16.8 %), having problems in transferring gambling payments (16.8 %), anticipating slim chances to win (16.1 %), having no extra money for the activity (10.7 %), having no access to Internet services (2 %), and assuming the activity was unsafe (2 %).

### Perceptions of Internet Gambling

Among the students who had not gambled online in the past year (n = 969), less than a third (28.9 %) regarded Internet gambling as a trendy, safe (19.9 %), and healthy entertainment (17.9 %). A considerable proportion perceived Internet gambling as potentially harmful (40.1 %) and more addictive than traditional forms of gambling (62 %). They supported strict governmental regulation and legislation (77.4 %).

Many online gamblers adopted a more positive attitude towards the activity. They perceived Internet gambling as a trendy (71.4 %), safe (54.3 %) and healthy entertainment (48.6 %) which provided an opportunity to win money (42.9 %). They disagreed that the activity was less regulated (57.1 %) and was potentially more addictive than traditional forms of gambling (57.1 %). Awareness of potential harms associated with Internet gambling was low (8.6 %).

Higher scores on the Perceptions of Internet Gambling Questionnaire (PIGQ) suggest a more positive attitude towards online gambling. The Internet gamblers scored significantly higher (mean score = 19.5) on the PIGQ than the non-Internet gamblers (mean score = 4.5) [(X_2_ (1, N = 638 = 11.7, *p* < 0.01)].

### Correlates of Problematic Internet Gambling

Using non-parametric Mann–Kendall Tau-b method (Table 2), problematic Internet gambling was significantly correlated with amount of money wagered, the male gender, Internet gambling frequency, early initiation, familial influence and senior school grades. The correlation coefficients were 0.380 (*p* < 0.001), 0.360 (*p* < 0.001), 0.338 (*p* < 0.001), 0.314 (*p* < 0.01), 0.298 (*p* < 0.01) and 0.219 (*p* < 0.5) respectively.

**Table 2:** Correlations between DSM-IV-MR-J scores and demographic, family and behavioral variables

Variables	DSM-IV scores
Gender	0.360***
School grades	0.219*
Money wagered each week	0.380***
Internet gambling frequency	0.338***
Early Internet gambling initiation	0.314**
Familial influence	0.298**

* *p* < 0.05, ** *p* < 0.01, *** *p* < 0.001 (two-tailed)

## Discussion

### Limitations of the Study

The study has several limitations. First, the use of a self-report survey questionnaire may cause comprehension, and memory mistakes. Second, the number of Internet gamblers was small, and there were only six female online gamblers. Hence, gender specific comparison and statistical analyses have been hampered. Future research should increase the sample size to obtain a sufficiently large number of Internet gamblers to identify the gender specific demographic and psychosocial characteristics of Internet problematic gamblers. Third, prudence is needed in generalizing survey findings which were generated from a small sample of Internet gamblers. Last, this is a cross-sectional study. The researcher did not attempt to identify causal relationships between problematic Internet gambling and the demographic or psychosocial variables. Future research may employ more vigorous methodologies to examine causal relationships.

### Internet Gambling Participation and Addiction

Replicating early research results (e.g., Griffiths 2001; Griffiths et al. 2009; McBride and Derevensky 2012), this survey shows that more adolescents gambled offline than online during the past year (63.5 vs. 3.5 %). The current 3.5 % online gambling participation rate falls within the range of 2–4.6 % found in local surveys (e.g., Hong Kong Polytechnic University 2002; University of Hong Kong 2005; Wong 2010b), but it is lower than the prevalence estimates reported in several recent western studies. For example, 24.3 % of 1,537 Iceland students (aged 13–18 years) wagered on the Internet during the previous 12 months (Olason et al. 2011). McBride and Derevensky (2012) noted 8 % of 465 Canadian college students (aged 18–20 years) gambled online.

Consistent with previous research findings (Griffiths and Barnes 2008; Matthews et al. 2009; Wood et al. 2007), this study supports the claim that the prevalence rates of problematic gambling are higher among online gamblers than offline gamblers. More than one-fifth (22.9 %) of the Chinese Internet gamblers surveyed were at-risk gamblers, and 5.7 % were probable pathological gamblers. Internet gamblers are 1.5 and 3.2 times more likely to develop pathological and at-risk gambling than offline gamblers.

This study found gender difference in online gambling involvement, confirming that Internet gambling is a male-dominated activity (82.9 % males vs. 17.1 % females) (Petry and Weinstock 2007; Romer 2010; Spectrum Gaming Group 2010; Wong 2010a). Boys were also more likely than girls to gamble excessively on the Internet (90 vs. 10 %) Ample research evidence indicates similar results (e.g., McBride and Derevensky 2012; Olason et al. 2011; Wong 2010a).

Difference in school grades was noted. The senior graders (grade 10–12) preferred gambling with money on the Internet, whereas more junior graders (grade 9–11) reported playing at the free trial sites. Senior graders may have more money available for gambling because adolescents of 15 years or above are eligible for legal employment. They are allowed to take up part-time jobs to earn pocket money. Parents also provide more pocket money to senior graders to support their growing school and daily expenses.

It is interesting to discover that many Internet gamblers (71.4 %) began playing on the free sites before wagering on the paid sites. It is likely that the free sites may become a “gateway” to subsequent gambling with money (McBride and Derevensky 2012). Since no age restriction is imposed on these practice sites, and no money is actually staked, teenagers may not perceive the activity as potentially risky or harmful (McBride and Derevensky 2012). On the contrary, many online gamblers surveyed perceived Internet gambling on both free and paid sites as a safe and healthy entertainment. Free sites do tempt the players to gamble for money. The potential perils of playing on these free sites are not known yet, and the relationship between the practice sites and the paid sites is unexamined (Byrne 2004; Derevensky and Gupta 2007; McBride and Derevensky 2012). Future research may explore these unstudied areas.

Majority (91.4 %) of the Internet gamblers staked at the paid sites well before 18 years, and 60 % made their first bet online before 12 years. Many were assisted to wager online and transfer gambling payment by family members (42.8 %) or friends (10.3 %). Replicating past research data (Jacobs 2000; Winters et al. 2002; Wong 2010a, b), early gambling initiation is found correlated with problem gambling. These findings indicate the need of early prevention prior to adolescence. Internet gambling awareness initiatives are hardly available currently in Hong Kong. It is necessary to develop school-based and public education programs to promote awareness of the temptation and potential risks associated with early and adolescent Internet gambling. Information about professional help and treatment services should also be provided. Adults, particularly family members should be warned against providing help in transferring Internet gambling payments for minors. Since 94.3 % of the young online gamblers wagered at home, strict parental supervision on children’s use of the Internet may deter underage online gambling at home.

Most of the participants (75.6 %) found it effortless to receive gambling information on the Internet. While 40 % of the online problem gamblers were tempted by attractive gambling advertisements, 37.1 % of the recreational gamblers were enticed by promotional offers of free play at the practice sites. This study indicates a serious lack of safeguards to protect underage and young students. Internet gambling providers should develop and enforce responsible gaming strategies to prohibit irresponsible advertising and promotional activities, and to prevent minors gamble online. Strict age and identity verification should be implemented, and warning messages could be included in all the advertisement and promotional materials.

Consistent with previous research data, the correlational results of this survey ratify problem gambling is associated with the male gender (Gupta and Derevensky 1998; Griffiths and Barnes 2008; Petry and Weinstock 2007), senior school grades (Wong 2010b), money wagered (McBride and Derevensky 2009; Nelson et al. 2008), gambling frequency (Vitaro et al. 2001; Wood et al. 2007), familial influence (Gupta and Derevensky 1997; Jacobs 2000), and early initiation (Jacobs 2000; Winters et al. 2002; Wong 2010a). Correlates are useful for identifying problematic Internet gamblers. Secondary prevention programs should target at these high-risk students.

Although this survey reveals that Internet gambling is still a minor activity among Hong Kong high school students, empirical evidence consistently indicates a trend of increasing Internet gambling among adolescents and young adults (Griffiths et al. 2009; McBride and Derevensky 2012; Olason et al. 2011). Since higher rates of problem gambling have been found among online gamblers than offline gamblers, there have been growing concerns about the potential deleterious effects of Internet gambling on the health of the public in general (LaBrie et al. 2008) and youth in particular (Derevensky and Gupta 2007; Jacobs 2000). More research is needed on this under-examined area to increase our understanding of this potentially more harmful activity.

## Conclusion

Studies on Internet gambling involvement and addiction among Asian adolescents are scarce (Wong 2010a). This survey provides evidence-based information on this unexamined research area. It enhances our understanding of how teenagers perceived Internet gambling, the reasons for participation and non-participation, and the correlates of problematic online gambling. The study also provides estimates of prevalence of Internet gambling involvement and addiction. The survey results have implications for prevention and future research. Currently, only a relatively small proportion of adolescents are engaging in Internet gambling. With the phenomenal growth of gambling sites, Internet gambling is likely to confer health risks for adolescent students because they are frequent Internet users who are not protected from irresponsible advertising and promotional activities. To safeguard adolescents from early gambling initiation and excessive gambling on the Internet, key stakeholders (e.g., the industry, the policy makers, parents of adolescent students, psychologists and health professionals) have to play a more active role in gambling-related awareness education and problem gambling prevention. More empirical research on Internet gambling is needed to further expand our understanding of the etiological factors for Internet gambling addiction. We need evidence-based data to inform policies, intervention and preventive strategies.
